# Grant Report on PREDICT-ADFTD: Multimodal Imaging Prediction of AD/FTD and Differential Diagnosis

**DOI:** 10.20900/jpbs.20190017

**Published:** 2019-10-30

**Authors:** Lei Wang, Ashley Heywood, Jane Stocks, Jinhyeong Bae, Da Ma, Karteek Popuri, Arthur W. Toga, Kejal Kantarci, Laurent Younes, Ian R. Mackenzie, Fengqing Zhang, Mirza Faisal Beg, Howard Rosen, Alzheimer’s Disease Neuroimaging Initiative

**Affiliations:** 1 Northwestern University Feinberg School of Medicine, Chicago, 60611 IL, USA; 2 School of Engineering Science, Simon Fraser University, Burnaby, V6A1S6 BC, Canada; 3 Keck School of Medicine of University of Southern California, Los Angeles, 90033 CA, USA; 4 Departments of Neurology and Radiology, Mayo Clinic, Rochester, 55905 MN, USA; 5 Department of Applied Mathematics and Statistics, Johns Hopkins University, Baltimore, 21218 MD, USA; 6 Department of Pathology and Lab Medicine, University of British Columbia, Vancouver, B6T1Z4 BC, Canada; 7 Department of Psychology, Drexel University, Philadelphia, 19104 PA, USA; 8 Department of Neurology, University of California, San Francisco, 94143 CA, USA; 9 Data used in preparation of this article were obtained from the Alzheimer’s Disease Neuroimaging Initiative (ADNI) database (http://adni.loni.usc.edu/). As such, the investigators within the ADNI contributed to the design and implementation of ADNI and/or provided data but did not participate in analysis or writing of this report. A complete listing of ADNI investigators can be found at: http://adni.loni.usc.edu/wp-content/uploads/how_to_apply/ADNIAcknowledgement_List.pdf

**Keywords:** dementia, Alzheimer’s Disease, brain imaging, FDG-PET, machine learning, Convolutional Neural Networks, morphometry, sex-differences, C9orf72

## Abstract

We report on the ongoing project “PREDICT-ADFTD: Multimodal Imaging Prediction of AD/FTD and Differential Diagnosis” describing completed and future work supported by this grant. This project is a multi-site, multi-study collaboration effort with research spanning seven sites across the US and Canada. The overall goal of the project is to study neurodegeneration within Alzheimer’s Disease, Frontotemporal Dementia, and related neurodegenerative disorders, using a variety of brain imaging and computational techniques to develop methods for the early and accurate prediction of disease and its course. The overarching goal of the project is to develop the earliest and most accurate biomarker that can differentiate clinical diagnoses to inform clinical trials and patient care. In its third year, this project has already completed several projects to achieve this goal, focusing on (1) structural MRI (2) machine learning and (3) FDG-PET and multimodal imaging. Studies utilizing structural MRI have identified key features of underlying pathology by studying hippocampal deformation that is unique to clinical diagnosis and also post-mortem confirmed neuropathology. Several machine learning experiments have shown high classification accuracy in the prediction of disease based on Convolutional Neural Networks utilizing MRI images as input. In addition, we have also achieved high accuracy in predicting conversion to DAT up to five years in the future. Further, we evaluated multimodal models that combine structural and FDG-PET imaging, in order to compare the predictive power of multimodal to unimodal models. Studies utilizing FDG-PET have shown significant predictive ability in the prediction and progression of disease.

## INTRODUCTION

Frontotemporal dementia (FTD), with the behavioral variant (bvFTD) being the most common form, is the leading cause of dementia in people under the age 60 [1], with an estimated prevalence of 15–22/100,000 [1–3]. Alzheimer’s dementia (AD) is the most common form of dementia in adults over the age of 65, affecting 5 million Americans (source: NINDS). Even though advances in imaging, pathology, and genetics research have improved our understanding of the underlying diseases [4–18], accurate antemortem diagnosis, as well as sensitive and specific biomarkers that can facilitate a differential diagnosis, remain elusive [19]. This is because specific neuropathologies are usually associated with a spectrum of clinical syndromes, and different clinical syndromes are related to multiple underlying neuropathologies [20,21]. For example, neuropathologies characteristic of Alzheimer’s disease are found in 15–30% of FTD patients [22], and significant numbers of AD cases carry neuropathological markers of frontotemporal lobar degeneration [23]. Because the success of disease-altering therapies depends largely on early and specific diagnosis, it is critical to develop sensitive and specific biomarkers for the underlying neuropathologies, which can then be used to identify the most appropriate patient populations for specific disease-altering interventions [24]. This project addresses the unmet need for antemortem biomarkers that can distinguish between dementias caused by different underlying neuropathologies.

PREDICT-ADFTD: Multimodal Imaging Prediction of AD/FTD and Differential Diagnosis is a four-year award, which is currently in its third year. This project was awarded in order to broadly study neurodegeneration using a variety of imaging and computational techniques so that we may be able to develop methods for the early and accurate prediction of disease and its course. The overarching goal of the project is to develop the earliest and most accurate biomarker that can differentiate clinical diagnoses. It is crucial to not only learn about FTD and AD, but related dementias in order to determine unique profiles of disease. The more known about typical onset, clinical presentation, neuropathology, including spatial location and temporal spread, genetic influence, and gross anatomy of each disease, the more we will be able to diagnose and treat in confidence. The following grant report will discuss several studies that have been conducted through the funding of this grant, ongoing studies, and future research based on these preliminary results.

### Overall Project Structure: A Multi-Site, Multi-Study Collaboration Effort

**Figure 1.**
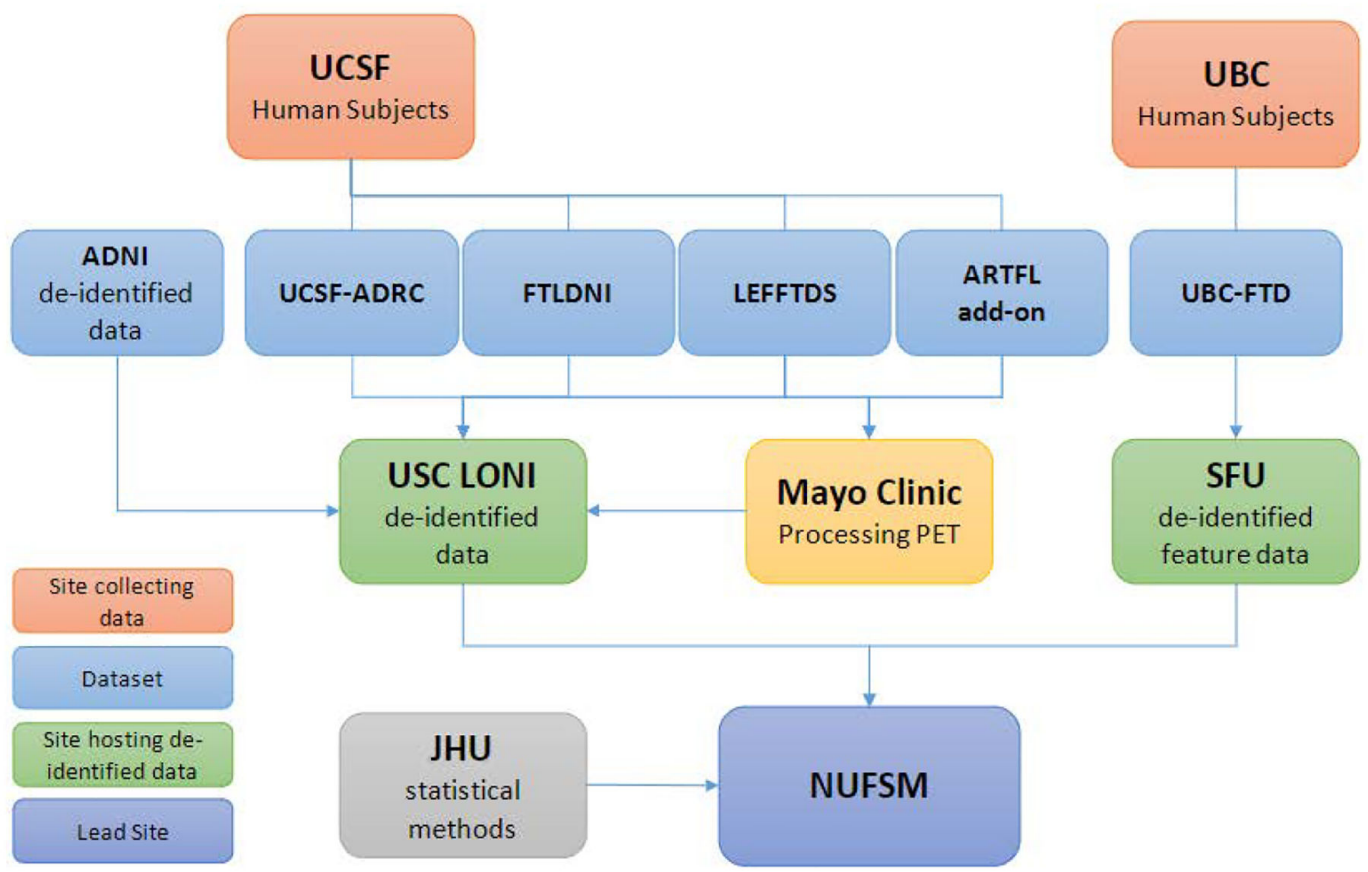
Graphical representation of study design. UCSF and UBC will act as main collecting sites which will distribute data for analysis and processing amongst collaborators. USCF: University of California San Francisco; UBC: University of British Columbia; ADNI: Alzheimer’s Disease Neuroimaging Initiative; UCSF-ADC: UCSF Alzheimer’s Disease Center; FTLDNI: Frontotemporal Lobar Degeneration Neuroimaging Initiative; LEFFTDS: Longitudinal Evaluation of Familial Frontotemporal Dementia Subjects; ARTFL: Advancing Research and Treatment for Frontotemporal Lobar Degeneration; UBC-FTD: UBC Frontotemporal Dementia; USC-LONI: University of Southern California Laboratory of Neuro Imaging; SFU: Simon Fraser University; JHU: Johns Hopkins University; NUFSM: Northwestern University Feinberg School of Medicine.

The funded project is widely collaborative, with research spanning seven sites across the US and Canada. These sites include Northwestern University Feinberg School of Medicine (NUFSM), Simon Frasier University (SFU), University of British Columbia (UBC), Johns Hopkins University (JHU), University of California at San Francisco (UCSF), The Mayo Clinic, and University of Southern California (USC). A graphical representation of the study design can be seen in Figure 1.

The lead site for the project is Northwestern University Feinberg School of Medicine under Principal Investigator Dr. Lei Wang. Dr. Wang developed a career that utilizes training in engineering, mathematics, and computer science to develop and apply computational anatomy tools to the analysis of multimodal neuroimaging datasets. His work exploits the use of multiple regions, multiple modalities, and multiple time points that can improve the understanding, tracking, and detection of these neuropsychiatric diseases. As PI and co-I on a number of NIH- and foundation- funded studies, Dr. Wang has established a track record of publication, collaboration, and leadership in these areas.

The M-PI for the project, Dr. Rosen, leads the University of California at San Francisco team. He is PI of the Frontotemporal Lobar Degeneration Neuroimaging Initiative (FTLDNI), leader of UCSF-Alzheimer Disease Research Center (ADRC) imaging core, MPI of Longitudinal Evaluation of Familial Frontotemporal Dementia (LEFFTDS), and administrative director and Project 2 (imaging) leader of the Advancing Research and Treatment for Frontotemporal Lobar Degeneration (ARTFL). Dr. Rosen will provide FTLDNI data, coordinate with Dr. Miller to provide the UCSF-ADRC data, and coordinate LEFFTDS data access. He will also coordinate the proposed ARTFL add-on scanning efforts.

Dr. Mackenzie from University of British Columbia will coordinate transferring of the UBC-FTD data to Dr. Beg at Simon Fraser University.

From Simon Frasier University, Dr. Faisal Beg has extensive experience in developing brain mapping algorithms and machine learning methods [25–28]. Drs. Wang and Beg have co-authored numerous peer-reviewed papers on neuroimaging biomarkers and machine learning methods for AD and FTD [25–27,29–42]. Dr. Beg will coordinate with the UBC team to perform image processing on the UBC-FTD dataset and send the computed data to Dr. Wang for machine learning analysis. Dr. Beg will continue to develop computational and classification methods for the proposed project.

As leader of the LEFFTDS MRI quality control (QC) team, Dr. Kantarci will supervise the Mayo Clinic personnel to use LEFFTDS procedures to perform imaging QC of the proposed ARTFL-add-on scans, and to upload the MRI data to University of Southern California Laboratory of Neuro Imaging (LONI).

Dr. Toga, as leader of the LEFFTDS data archive team, will supervise the USC personnel on archiving the ARTFL-add-on scans and on making the data available to the study team.

Dr. Younes from Johns Hopkins University has made significant contributions in the area of statistical modeling of longitudinal change associated with neurodegeneration [43–47]. Dr. Younes will assist with longitudinal analysis of neuroimaging data and on various statistical approaches to integrating data across different platforms.

ADNI Data: ADNI data used in the preparation of this article were obtained from the Alzheimer’s Disease Neuroimaging Initiative (ADNI) database (adni.loni.usc.edu). The ADNI was launched in 2003 as a public-private partnership, led by Principal Investigator Michael W. Weiner, MD. The primary goal of ADNI has been to test whether serial magnetic resonance imaging (MRI), positron emission tomography (PET), other biological markers, and clinical and neuropsychological assessment can be combined to measure the progression of mild cognitive impairment (MCI) and early Alzheimer’s disease (AD).

### Aims of the Grant

#### Aim 1a.

Use cross-sectional structural MRI to develop predictive biomarker models for differentiating bvFTD from AD and NC. We hypothesize that our machine learning approach can capture the spatial distribution patterns of structural features characteristic of different neuropathologies, and that they can be combined with clinical/cognitive features to distinguish between bvFTD and AD. Aim 1b. Include longitudinal structural imaging data to increase predictive power.

#### Aim 2a.

Use cross-sectional FDG-PET imaging to develop predictive biomarker models for differentiating bvFTD from AD and NC. We hypothesize that our machine learning approach can capture the spatial distribution patterns of FDG-PET features characteristic of different neuropathologies, and that they can be combined with clinical/cognitive features to distinguish between bvFTD and AD. Aim 2b. Include longitudinal FDG-PET imaging data to increase predictive power.

#### Aim 3.

Evaluate multimodal models that combine structural and FDG-PET imaging and compare the predictive power of multimodal and unimodal models. We hypothesize that the multimodal model will have higher predictive power.

## STRUCTURAL MRI STUDIES ON ANTEMORTEM BIOMARKERS

### Background

Significant progress has been made in the development of biomarkers for early detection of dementia. However, distinguishing between different types of underlying pathological disease states remains a challenge [19]. Due to neuropathological heterogeneity within clinical diagnoses, accurate antemortem diagnosis is difficult [20, 21]. In addition, several neuropathologies, *i.e*., “mixed dementia” may exist in the same person, even in the same region of the brain. Identifying specific protein aggregates in an individual’s brain is currently done through postmortem autopsy. The success of any future treatments for neurodegenerative disorders will depend largely on the ability to achieve an early and accurate antemortem diagnosis.

Computational analysis of antemortem structural magnetic resonance imaging (MRI) data provides a minimally invasive approach to identifying subtle changes in brain shape of living subjects [48]. Multiple studies have employed structural MRI in an attempt to classify dementia based on patterns of atrophy, but few can confirm their results with pathological evidence [49,50]. Therefore, it is of critical importance to utilize antemortem structural MRI in order to develop early and accurate biomarkers of postmortem disease.

Additionally, due to the prevalence of “mixed dementia” and the heterogeneity seen in FTDs, studying population with specific genetic risks may elucidate differences between measurable effects of neuropathologies in the antemortem brain. Two groups of mutation carriers, GRN and C9orf27 have been identified in their importance with familial onset FTD, accounting for a significant portion of familial carriers. Both these groups show increased deposition of the transactive response DNA binding protein of 43 kDa (TDP-43): a key neuropathology within FTD. Therefore, studying patients who are known carriers, as well as non-carriers, will allow for the study of the genetic influence on the deposition of neuropathology and the specific burden that TDP-43 places on the brain.

### Progress Report

#### Study 1: Hippocampal subfield deformity patterns for subtyping Non-semantic primary progressive aphasia (PPA)

Using methods developed by Dr. Wang and colleagues, FreeSurfer-initiated Large-Deformation Diffeomorphic Metric Mapping (FSLDDMM) [31,38,40] hippocampal shape maps were created from structural MR in 37 non-semantic PPA subjects, 15 individuals with early dementia of the Alzheimer type (aMCI/DAT), and 32 healthy controls. The goal of this project was to identify subgroups who cluster based on probable neuropathology. A two-step, semi-supervised statistical learning procedure was used to classify PPA participants into subgroups based on hippocampal shape measures and WMS-III memory performance. Hippocampal shape scores and WMS-III memory scores were entered into a logistic regression procedure to produce a model set of features. In the second, unsupervised step, we utilized k-means and hierarchical clustering on the model set on all PPA patients to discover subgroups. Stability of subgroup membership was evaluated with alternative classification methods: without training sets, with a training set of only DAT subjects, and using a single shape score. Finally, we compared the resulting subgroups to determine relationships between memory and executive function. Two PPA subgroups (*n* = 24 and 13) were found, one presumed to carry AD neuropathology (PPA-ADN) and one representative of a mixture of non-AD neuropathologies including FTLD (PPA-NonADN). Subgroup membership was robust across alternative methods. The PPA-ADN subgroup displayed common AD characteristics, including greater memory dysfunction and lower semantic fluency. PPA-NonADN showed characteristics commonly associated with non-AD neuropathology, including greater working memory dysfunction and the early preservation of memory. Further, hippocampal subfield atrophy in PPA-ADN resembled aMCI/DAT patterns. These findings are consistent with our prior findings in patients with autopsy-confirmed PPA with AD neuropathology. This work was published in *Alzheimer’s & Dementia* [40].

#### Study 2: Rush University Alzheimer’s Disease Center antemortem hippocampal surface co-registration with postmortem neuropathology

Center (RADC), we obtained ante-mortem T1-weighted structural MRI scans and quantitative post-mortem measures for β-amyloid, PHF tau tangles, and TDP-43 by immunohisto-chemistry from 42 subjects (mean age at imaging 87.6 years, mean imaging-death interval 2.7 years), and generated hippocampal surfaces using FSLDDMM [31,38,40]. We related the neuropathological measures to the vertex-wise hippocampal surface displacement measures (relative to a reference mean) using linear mixed-effects model. Through a collaboration with the Rush University Alzheimer’s Disease.

**Figure 2.**
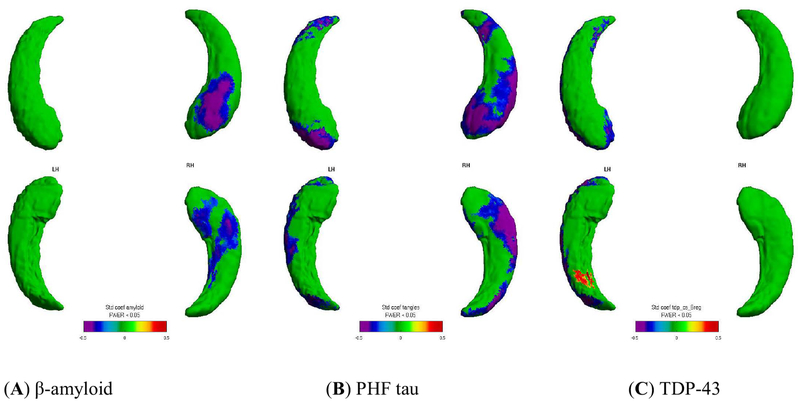
Visualization of the relationship between global immunohistochemical pathology burden and hippocampal surface deformity. Spatial distribution patterns for relating hippocampal surface deformities to immunohistochemistry: (**A**) β-amyloid, (**B**) PHF tau, (**C**) TDP-43. Bluish colors visualize negative associations between higher neuropathology with more inward surface deformity (*i.e*., localized volume loss). Left hippocampus is on the left side of the figure. Panels show results of univariate analysis.

Significant (FWER < 0.05, random field theory) [51,52] associations were visualized onto the surface vertices for localization. We found that higher measures of β-amyloid, PHF tau tangles, and TDP-43 inclusions were all related to increased inward surface deformity (*i.e*., local volume loss). Further, the neuropathologies were related to distinct spatial distribution patterns (Figure 2), and the patterns for the AD neuropathologies generally followed the CA1, subiculum subfields. This work was published in *Neurobiology of Aging* [53].

#### Study 3: Gray matter changes in asymptomatic C9orf72 and GRN mutation carriers

Clinically asymptomatic subjects from families with C9orf72 mutation (15 mutation carriers, C9orf72+; and 23 non-carriers, C9orf72−) and GRN mutations (9 mutation carriers, GRN+; and 15 non-carriers, GRN−) underwent structural neuroimaging (MRI). Cortical thickness and subcortical gray matter volumes were calculated using FreeSurfer. Group differences were evaluated, correcting for age, sex and years to mean age of disease onset within the subject’s family. The C9orf72+ group exhibited cortical thinning in the temporal, parietal, and frontal regions, as well as reduced volumes of bilateral thalamus and left caudate compared to the entire group of mutation non-carriers (NC: C9orf72− and GRN− combined). In contrast, the GRN+ group did not show any significant differences compared to NC. C9orf72 mutation carriers demonstrate a pattern of reduced gray matter on MRI prior to symptom onset compared to GRN mutation carriers. These findings suggest that the preclinical course of FTD differs depending on the genetic basis and that the choice of neuroimaging biomarkers for FTD may need to take into account the specific genes involved in causing the disease. This work was published in *NeuroImage: Clinical* [54].

#### Study 4: Sex differences within healthy subjects utilizing structural MR and APOE4 status from ADNI

**Figure 3.**
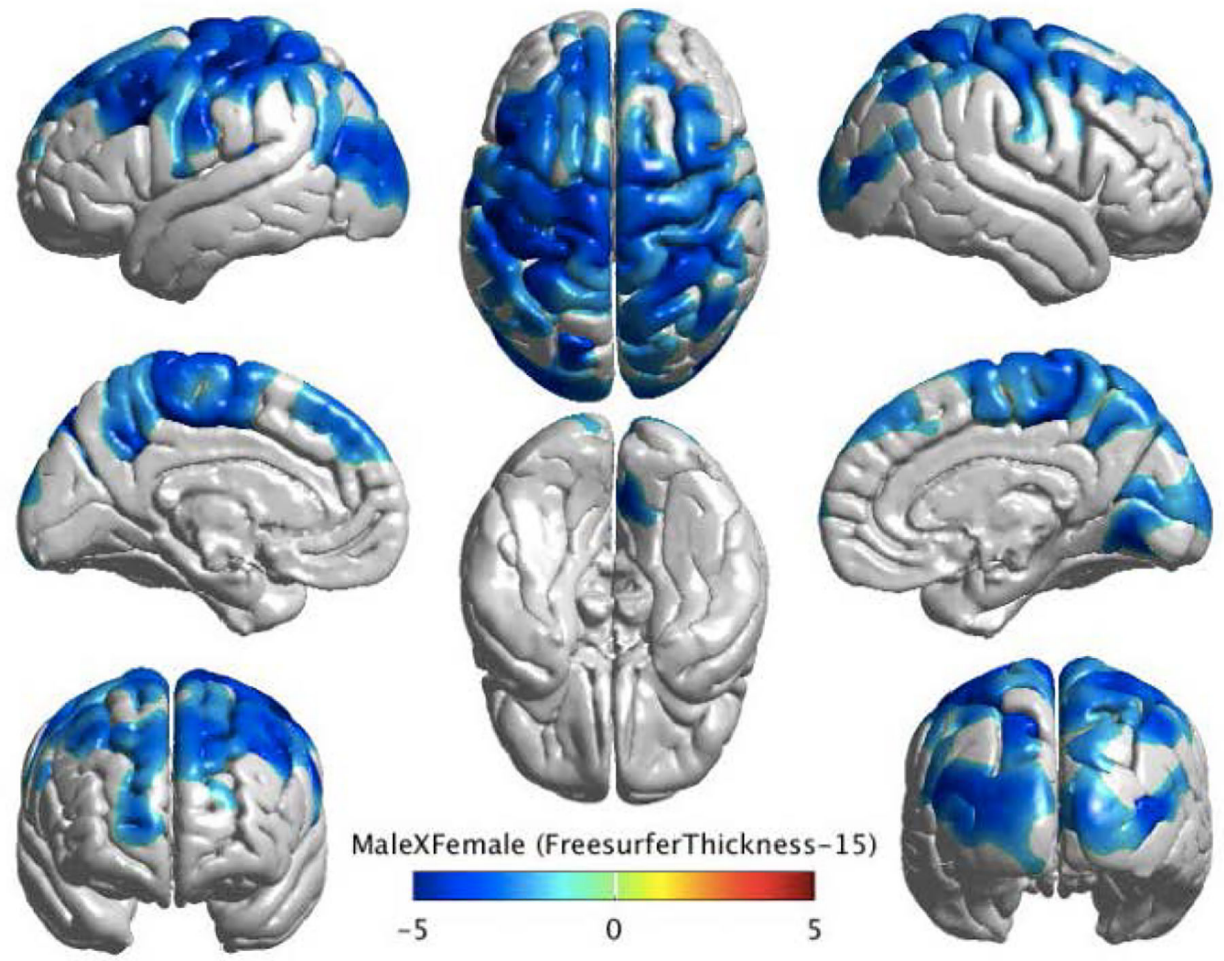
The figure above shows the cortical thickness map for a group difference between males and females. Cortical thickness has been analyzed for 738 subjects (ADNI and AIBL combined) and controlled for age, APOE4, intracranial volume (ICV), field strength, and scanner. The *p*-values have been corrected for multiple comparisons using random field theory. The figure demonstrates that males have a thinner cortex than females, as indicated by a negative *t*-statistic in blue.

**Figure 4.**
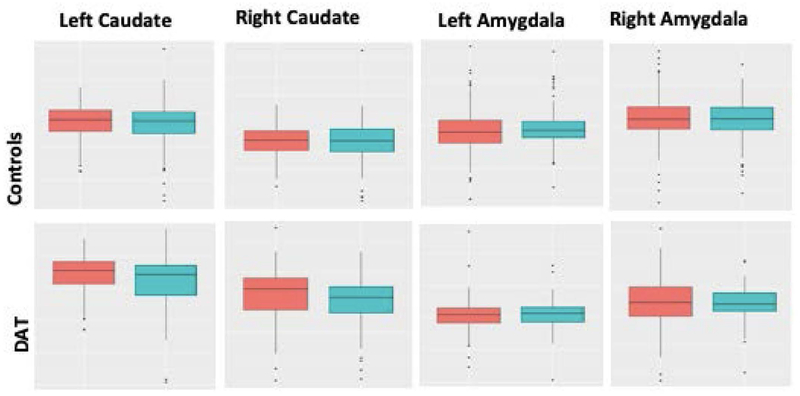
Rates of atrophy over two years. Males (**blue**) and females (**red**) in control and DAT group. For bilateral Caudate and amygdala, male brains showed greater atrophy in the control group, whereas females had greater atrophy in the DAT group. The *t*-statistic provided the extent of atrophy on both groups.

In the interest of improving predictive diagnosis, it is pressing that we have a more nuanced understanding of individual factors contributing to similarities and differences in brain structure across subjects [55]. To this aim, we set out to gain a more detailed picture of how the brains of males and females differ, given the known discrepancy in the prevalence of neurodegenerative disorders between the sexes [56]. To this aim, we examined differences in brain volumes for 742 cognitively normal healthy subjects in the ADNI and Australian Imaging Biomarkers and Lifestyle flagship study of aging (AIBL) databases. Additionally, we explored the effect of APOE4 gene status on brain volume differences in healthy subjects. Regression methods using general linear models (GLM) were used to remove the effect of various covariates on the structural volume to ensure an unbiased statistical evaluation. Controlling for the effect of age, intracranial volume, field strength, and scanner type, results showed significant volume differences in cortical and subcortical structures including the bilateral amygdala and cerebellum (Figures 3 and 4).

All other brain structure volumes showed no significant differences between genders. Further, the existence of APOE4 gene does not alter the volume differences between healthy male and female subjects. Further longitudinal analyses revealed a greater decrease in volumes over time for males in the healthy group, and greater decreases over time for females in the AD group.

### Planned Research

Currently, results have been replicated from Study 2 on the original 42 subjects and we plan to expand the sample to 129 subjects. Future research may include expanding to other subcortical regions such as amygdala and basal ganglia. Further, once a disease specific atlas is developed, we hope to compare this to other populations such as a sample from ADNI. There are currently 64 ADNI subjects who have undergone autopsy, and we plan to replicate methods from Study 2 in this population and compare the deformation atlases between the populations.

### Papers and Presentations

- Christensen A, Alpert K, Rogalski E, Cobia D, Rao J, Beg MF, *et al.* Hippocampal subfield surface deformity in nonsemantic primary progressive aphasia. Alzheimers Dement (Amst). 2015;1(1):14–23 [40].
- Popuri K, Dowds E, Beg MF, Balachandar R, Bhalla M, Jacova C, *et al*. Gray matter changes in asymptomatic C9orf72 and GRN mutation carriers. Neuroimage Clin. 2018;18:591–598. doi: 10.1016/j.nicl.2018.02.017 [54].
- Hanko V, Apple AC, Alpert KI, Warren KN, Schneider JA, Arfanakis K, *et al*. *In vivo* hippocampal subfield shape related to TDP-43, amyloid beta, and tau pathologies. Neurobiol Aging. 2019;74:171–181. doi: 10.1016/j.neurobiolaging.2018.10.013 [53].
- Sangha O, Stocks J, Popuri K, Wang L, Beg MF. Longitudinal Sex Differences in Gray Matter Atrophy for Alzheimer’s Disease. Presented at the Alzheimer’s Association International Conference; 14–18 July 2019; Los Angeles, CA, USA [57].

## STUDIES ON MACHINE LEARNING

### Background

Machine learning has been implemented in a variety of clinical research [58,59], including AD and FTD. In 2014, Li *et al.* [60] achieved 74.8% classification accuracy in predicting AD conversion from MCI by using a hierarchical interaction model. Basaia *et al.* [61] implemented a deep Convolutional Neural Network and produced 74.9% accuracy. However, the previous research did not fully validate their results based on current machine learning standards of the field. Davatzikos *et al.* [62] used principle component analysis to select features from MRI scans and resulted in 84.3% classification accuracy in distinguishing AD from FTD. However, these studies involved small sample sizes and did not consider longitudinal trajectories. The large amount of neuroimaging data available through our multi-site consortia made it ideal for machine learning approaches that could lead to accurate prediction of disease progression and distinction between AD and FTD.

### Progress Report

#### Study 1: Assessing the goodness of harmonization for combining data from multiple sites

**Figure 5.**
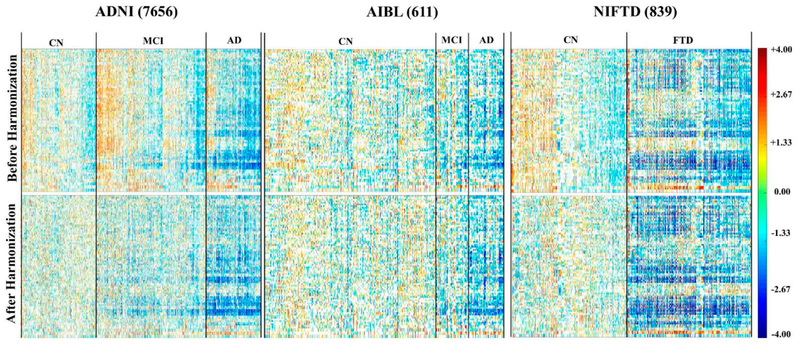
Zscape shows a panoramic view of all the structures across the entire datasets. The top and bottom panels show the standardized variation before and after data harmonization for 4 different datasets: ADNI (7656 images) [64], AIBL (611 images) [65], NIFTD (839 images) [66], and Parkinson’s Progression Markers Initiative PPMI (1507 images) [67]. Data are first categorized into diagnosis groups, with the normal healthy group as the reference control group to calculate the *Z*-score. Each diagnostic group is then by gender, which are further divided into 3T and 1.5T subgroups. The color spectrum from red to blue shows the value of the Z-score decreased from +6 to −6, representing the level of volume shift from the mean of the reference control group.

When pooling datasets from large multicenter databases, the measured data are affected by multiple confounding covariates, which in turn increase the data variability, thereby hinder the ability of any machine-learning-based classifiers to detect the actual effect of interest, such as changes due to the disease. Therefore, it is important to “harmonize” the data to remove the effect of various covariates. We have investigated efficient ways to evaluate the “goodness” of covariates harmonization. We analyzed multi-site, longitudinal MR image datasets acquired in different cohorts. We evaluated the distribution of brain structure volumes over these datasets before and after accounting for multiple covariates such as total intracranial volume, scanner field strength, sex, and age. Two techniques were used: (1) the empirical cumulative distribution function and (2) A panoramic visualization of the standard variation over the entire datasets–Zscape (Figure 5). This work was published in *Human Brain Mapping* [63].

#### Study 2: Novel classification system of AD

**Figure 6.**
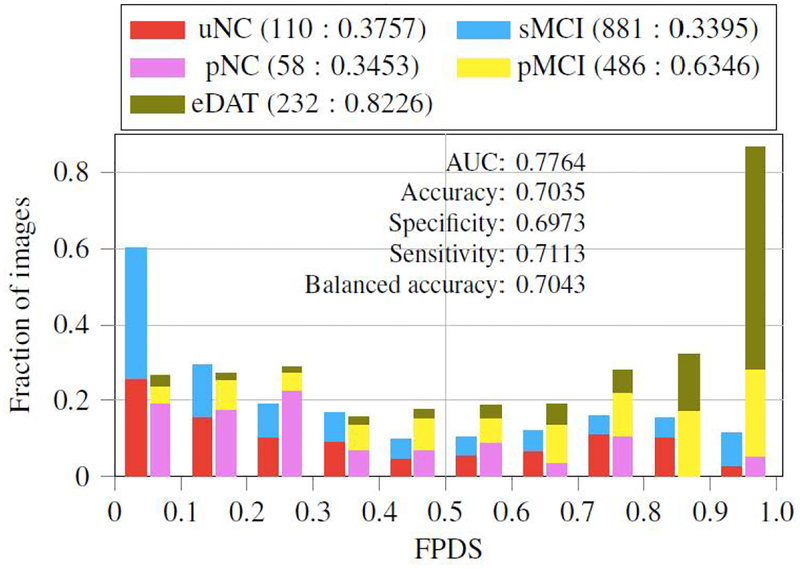
Distribution and classification power of FDG-PET DAT score (FPDS) as a biomarker. FPDS can successfully classify a multiple AD trajectories. For example, eDAT and pMCI patients tend to have a higher FPDS score while the rest of patients have a lower score. Its AUC classification resulted in 77.64%. uNC = Unstable normal control; pNC = Progressive normal control; eDAT = Early DAT; sMCI = Stable MCI; pMCI = Progressive MCI.

We constructed novel stratification categorizing dementia of AD type (DAT) patients to 7 trajectories based on their initial and final diagnosis in the past. We again grouped them into two sets, DAT+, whose clinical status progressed to AD, and DAT−, whose clinical status did not progress to AD. Then we implemented an ensemble of probabilistic multiple-kernel learning classifiers for classifying DAT− and DAT+. The classifiers were trained on baseline images taken from 360 sNC (representing the DAT− class) and 238 sDAT (representing the DAT+ class) subjects. The classifiers were then tested on 110 uNC (unstable normal control), 58 pNC (progressive normal control), 232 eDAT (early DAT), 881 sMCI (stable MCI) and 486 pMCI (progressive MCI) images that included longitudinal imaging data. The overall classification accuracy was 78% by AUC. Subsequently, the supervised machine learning framework produced a continuous value between 0 and 1, termed as the FDG-PET DAT score (FPDS), indicating the probability of the subject’s FDG-PET measure to be belonging to the DAT trajectory, *i.e*., how likely is the subject to be clinically diagnosed with DAT (Figure 6). Furthermore, we tested our model in predicting AD conversion in 2, 3, and 5 years and obtained 81%, 80%, and 77% classification AUC. With this result, we were able to confirm that our model produced a reliable biomarker that can distinguish patients who are progressive to AD and who are not. This work was published in *NeuroImage: Clinical* [68].

#### Study 3: Predicting conversion to AD from MCI

**Figure 7.**
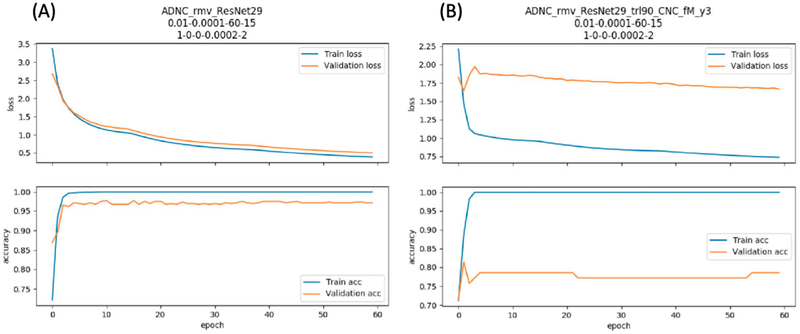
Performance of Residual Network 29. (**A**) Classifying AD from NC as a source task. (**B**) Transfer learning model on classifying sMCI from pMCI.

The third project proposed to predict conversion from MCI or NC to AD. We implemented a novel deep learning method, the Residual Network, in classifying patients who would progress to AD (pMCI) in at most 3 years and who would stay in MCI (sMCI) for at least 3 years. We firstly conducted a source task which classified NC *vs* AD with more than 2500 MRI scans from ADNI and obtained 97% classification accuracy within an independent test set. Then we transferred this domain knowledge in classifying sMCI *vs* pMCI and obtained 81% classification accuracy, which is the state-of-the-art performance. With this study, we were able to improve the performance of an AD conversion predicting model and revisited its potential in clinical practice. Figure 7 shows results from our deep learning performance on source task transfer learning.

#### Study 4: Multiclass modeling of FTD subtypes using Relevance Vector Machine

This last project aimed to build a multi-classification model classifying 5 FTD subtypes and AD, which are clinically similar enough to be misdiagnosed. We used 821 subjects’ MRI scans in total and implemented Relevance Vector Machine (RVM) as a classification method. The result was successful as the confusion matrix in Table 1 presents. We fully validated our results by using 5-fold inner cross validation and 3-fold outer cross validation.

Furthermore, we also implemented RVM in predicting AD pathology in a cohort of subjects diagnosed with PPA. Based on whole brain and regions of interest as a training resource, it resulted in 98% classification accuracy utilizing gray and white matter scans as inputs. With these results, we confirmed the potential application of computer-aided diagnosis system.

### Planned Research

In future work, we will further augment the data from updated ADNI and LEFFTDS data storage and improve our models’ performance. Also, previous machine learning methods lack interpretability as they cannot validate the features that were recognized by models during the training process. Therefore, we plan to provide a reasonable explanation of our prediction accuracy by implementing a gradient Class Activation Map which can visualize the features of our model. Such a study will use the weight metrics from the machine learning algorithms to identify which areas of the brain are more important in the classification of disease type. Using this, we will be able to identify regions of interest that may have not been previously identified or may also provide crucial confirmation of significant brain regions within FTDs.

### Papers and Presentations

- Popuri K, Balachandar R, Alpert K, Lu D, Bhalla M, Mackenzie IR, *et al*. Development and validation of a novel dementia of Alzheimer’s type (DAT) score based on metabolism FDG-PET imaging. NeuroImage Clin. 2018;18:802–13 [68].
- Ma D, Popuri K, Bhalla M, Sangha O, Lu D, Cao J, *et al*. Quantitative assessment of field strength, total intracranial volume, sex, and age effects on the goodness of harmonization for volumetric analysis on the ADNI database. Hum Brain Mapp. 2019;40(5):1507–1527. doi: 10.1002/hbm.24463 [63].
- Bae J, Heywood A, Stocks J, Jung Y, Popuri K, Beg M, *et al.* End-to-end 3D-Convolutional Neural Network for Predicting Conversion from Mild Cognitive Impairment (MCI) to Alzheimer’s Dementia (AD). Presented at the Alzheimer’s Association International Conference; 14–18 July 2019; Los Angeles, CA, USA [69].
- Bae J, Heywood A, Stocks J, Jung Y, Popuri K, Beg M, *et al.* End-to-end 3D-Convolutional Neural Network, Presented at the Society for Neuroscience Conference; 19–23 October 2019; Chicago, IL, USA [70].

## STUDIES ON FDG-PET AND MULTIMODAL/LONGITUDINAL ANALYSIS

### Background

In Aim 2a/b, we aspired to use cross-sectional and longitudinal FDG-PET imaging to develop predictive biomarker models for differentiating bvFTD from AD and NC. There is an increasing clinical use of functional imaging biomarkers for the differential diagnosis of early-stage of neurodegenerative disease. In the research diagnostic criteria proposed by the International Working Group [71] and in the recommendations of the National Institute on Aging–Alzheimer’s Association (NIA-AA) [72–74], biomarkers are defined as an expression of pathophysiological aspects of disease, and are indicated for use to increase confidence in the diagnosis. Furthermore, these biomarkers are increasingly used in clinical trials for subject selection and stratification, safety and proof-of-concept assessments, and monitoring of treatment effects [75,76]. ^18^F-Fluorodeoxyglucose positron emission tomography (FDG-PET), which reflects glucose metabolism mainly from neurons [77], has been extensively evaluated in the frame of early-stage neurodegeneration, showing good accuracy in identifying patients who later convert to AD dementia [78–81]. Additionally, FDG-PET has been approved by the US Medicare health insurance program for diagnosis of FTD, and has been shown to increase diagnostic accuracy beyond that of clinical features alone when differentiating between FTD and AD [82].

### Progress Report

#### Study 1: Using FDG-PET to identify antemortem biomarkers of progression to AD

To hone the predictive validity of FDG-PET within AD patients alone, we employed multi-state Markov transition models, as well as multi-level models on three classes of patients: NC, MCI, and AD. This work was completed in the interest of developing more sensitive ante-mortem biomarkers for progression of MCI to AD-Dementia. More precise delineation of the areas particularly sensitive to neurodegeneration caused by AD allow our diagnostic tools more accuracy for distinguishing AD from other neurodegenerative diseases, including FTD. Multi-state modeling is a state-transition modeling approach based on Markov processes whereby individual patient-level data is used to build survival regression models for estimating rates of transition between stages of disease. To this aim, we utilized FDG-PET data available from the Alzheimer’s Disease Neuroimaging Initiative (ADNI). Specifically, we used standardized uptake value ratio (SUVR) features calculated from 147 patches from baseline to 96 months to predict transition probability from one state to another over time. The developed models were able to consistently identify several brain regions as significant predictors of conversion. Significant predictors in the model include right hippocampus (*p* < 0.001), left entorhinal cortex (*p* = 0.008), left isthmus of cingulate gyrus (*p* = 0.011), left precuneus (*p* = 0.058), and right medial temporal lobe (*p* = 0.028). Additionally, independent of FDG-PET, Apoe-4 status remained a significant predictor in the model (*p* = 0.017). The presented models identify key features in the prediction of progression from MCI to AD, and extend to show significant predictive ability of deficiencies in brain glucose metabolism. Future work in this domain will employ similar state-of-the-art transitional models to address the discrepancy in transition across additional variants of neurodegenerative diseases, including FTD.

#### Study 2: Multimodal comparison of neurodegeneration using ADNI FDG-PET to identify differences within AD and MCI

Neurodegeneration caused by pathological protein aggregation in AD and FTD is at least partly reflected in both gray matter cortical atrophy and glucose hypometabolism. In Aim 3 of the grant, we proposed to evaluate multimodal models that combine structural and FDG-PET imaging and compare the predictive power of multimodal to unimodal models. It has been suggested that abnormalities on FDG-PET may occur before structural changes in the brain in AD [83,84]. More specifically, comparison of structural and metabolic reductions show that atrophy begins in the medial temporal lobes, whereas metabolic changes occur in the posterior cingulate gyri and parietal lobule [85]. To date, researchers do not understand why neurodegeneration is reflected in disparate spatial and temporal patterns between structural and functional imaging modalities, nor the relationship of these variations to clinical presentation or demographics. To this aim, we investigated the relationship between brain structure and function and whether concordance between metrics varies by clinical presentation. We used structural and functional brain images from ADNI, and computed W-score maps that adjusts for the effect of normal aging. Pearson correlations were computed per individual across 68 Freesurfer-ROIs [86] of atrophy and hypometabolism, reflecting individual consistency in the degree of atrophy and hypometabolism across the entire brain. To investigate the discriminatory power of our multimodal data and compare to unimodal approaches, logistic regression assessed whether a whole-brain correlation score is predicative of later conversion to DAT, and whether this effect was greater than any imaging modality alone. For this analysis, 132 progressive MCI patients with a structural and functional scan at a time point 12 months before conversion to DAT were compared to a representative sample of 132 stable MCI patients.

**Figure 8.**
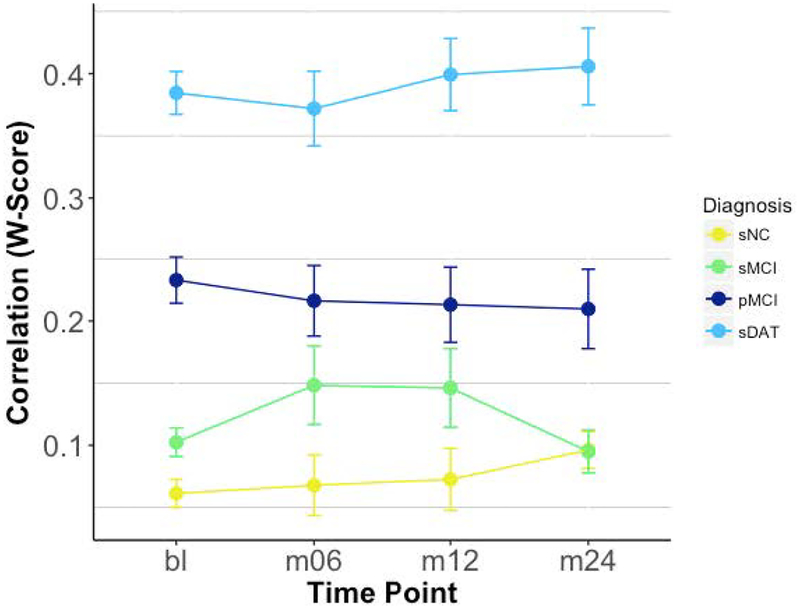
Whole-Brain Individual Correlations by Time & Diagnosis. sNC (stable normal control), sMCI (stable Mild Cognitive Impairment), pMCI (progressive Mild Cognitive Impairment), sDAT (stable dementia of Alzheimer’s type).

Results of the logistic regression indicated that there was a significant effect of correlation score and APOE4-status on conversion probability. Further, we found that individuals at more severe disease stages had much higher degrees of whole-brain consistency in degree of atrophy and hypometabolism (Figure 8).

#### Study 3: deep-neural-network-based Alzheimer’s Dementia score using 3D FDG-PET image only, as well as associated MRI

Finally, the AD-related functional and structural pathological changes are a continuous, progressive process. To achieve early diagnosis of AD using FDG-PET, it is important to predict such continuum directly from the patterns of metabolism alterations extracted from the correlated FDG-PET brain images. Firstly, we constructed a 3D convolutional neural network with residual connections that translate the FDG-PET images into a single probability score to represent the AD pathology continuum. The model weights of this network were firstly trained on subjects that are longitudinally stably diagnosed as normal control and DAT, achieving AUC of 0.976 with five-folds cross-validation. Testing of the training results on an independent test set showed AUC of 0.881 to predict the conversion of MCI to DAT within 3 years of conversion time. Our method also produced saliency and class activation maps localizing DAT-affected brain regions that are responsible for distinguishing converters from non-converters.

Furthermore, we also investigated a multi-scale, multi-modal deep neural network to learn the AD-pathology-induced metabolism pattern alterations. Similar to the approach in the previous study, we also performed comprehensive validation on FDG-PET data taken from 1051 subjects, demonstrating great generalization capability. Our results showed that the ensemble of multiple classifiers improved the stability and robustness of the classification performance. This work was published in *Medical Image Analysis* [87].

### Planned Research

Future work in this domain will examine whether region-specific correlations may offer even greater discriminatory power and allow for the discovery of inter-modality topographic discrepancies that can shed light on spatial or temporal discordance observed between imaging modalities in AD. Continued research will examine regional correlations between cortical atrophy and glucose hypometabolism which can serve as a biomarker for disease severity, and be used to predict conversion to DAT in MCI subjects. Further, the early and accurate diagnosis of Alzheimer’s disease is impeded by the high volume of MCI patients who present with atypical or non-amnestic neuropsychological profiles [88]. Research examining disease progression in MCI must be equipped to explain or account for heterogeneity in clinical or cognitive presentation. In MCI, differing cognitive profiles are multi-determined, but may reflect differing pathological processes that can be more clearly elucidated with multi-modal approaches. Additional research under Aim 3 will examine distinct cognitive trajectories of MCI using the multi-modal integration techniques. Finally, current research examining functional and structural alterations in FTD have largely done so separately, lacking the ability to explore the relationships between these changes. In particular, functional alterations concordant or discordant to structural changes or vice versa might provide valuable information for accurate diagnosis and staging of the FTD. Further research under Aim 3 will assess the multimodal integration and correlation among FTD subjects, and how that might offer greater discriminatory power between AD and FTD.

### Papers and Presentations

- Zhang F, Niu X, Heywood A, Stocks J, Beg MF, Wang L. Using Multi-state Markov Transition Models and Multilevel Models to Identify Biomarkers of AD using ADNI FDG-PET data. Presented at the Alzheimer’s Association International Conference; 14–18 July 2019; Los Angeles, CA, USA [89].
- Stocks J, Bae J, Sangha O, Popuri K, Beg MF, Wang L. The Relationship between Cortical Neurodegeneration and FDG-PET Hypometabolism as a Disease Marker Across Stages of Alzheimer’s Dementia. Presented at the Alzheimer’s Association International Conference; 14–18 July 2019; Los Angeles, CA, USA [90].
- Lu D, Popuri K, Ding GW, Balachandar R, Beg MF. Alzheimer’s Disease Neuroimaging Initiative. Multiscale deep neural network based analysis of FDG-PET images for the early diagnosis of Alzheimer’s disease. Med Image Anal. 2018;46:26–34. doi: 10.1016/j.media.2018.02.002 [87].

## DISCUSSION

We have presented a multitude of research projects both completed and in progress that are a product of the highly collaborative, multi-site, multi-study project presented here. Recent advances in imaging, pathology, and genetics have improved our understanding of FTD. Despite these advances, sensitive and specific biomarkers that can be used to facilitate an antemortem diagnosis remain elusive. Two decades of clinical trials of dementia patients have failed to produce effective disease-modifying drugs [91], and inaccurate antemortem diagnosis has been implicated as a key contributing factor. As potential disease-modifying treatments are being developed, sensitive and specific biomarkers will be needed, so that they can be used to identify the most appropriate patient populations. In this project, we are addressing the unmet need for antemortem biomarkers that can identify patients with specific neuropathologies, so that future disease-altering drugs can be tested with more success. Therefore, it is crucial to continue to advance the field utilizing multi-modal systems for research strategies.

The advent of large-scale consortia databases such as ADNI combined with rapid technical advancements in machine learning and statistical modeling, have significantly advanced the development of neuroimaging biomarkers for AD and related dementias. Because of such possibilities, we presented several studies on structural MRI, FDG-PET, and machine learning. With the development of methodologies to map surface deformation of subcortical structures, we were able to visualize and quantify areas of the hippocampus that are unique to underlying neuropathology. Our studies on FDG-PET revealed key features in the progression of disease. And through state-of-the-art machine learning algorithms, we can accurately classify and predict the progression of disease.

The research presented here provides crucial information on our understanding of disease. This work is critical in numerous ways. The accurate diagnosis of disease is the first step in providing the best treatments possible to affected individuals. Further, the accurate prediction of disease progression is an invaluable tool in patient care. In its third year, this project has already contributed greatly to body of literature within FTD and AD. However, research in progress to be completed in the final year of the grant stand to enhance the literature even further.

## CONCLUSIONS

We report on the multi-site, multi-study collaboration project “PREDICT-ADFTD: Multimodal Imaging Prediction of AD/FTD and Differential Diagnosis,” describing completed and future work supported by this grant. This project has completed a number of projects, focusing on (1) structural MRI; (2) machine learning; and (3) FDG-PET and multimodal imaging. Studies utilizing structural MRI have identified key features of underlying pathology by studying hippocampal deformation that is unique to clinical diagnosis and also post-mortem confirmed neuropathology. Several machine learning experiments have shown high classification accuracy in the prediction of disease based on Convolutional Neural Networks utilizing MRI images as input. In addition, we have also achieved high accuracy in predicting conversion to DAT up to five years in the future. Further we evaluated multimodal models that combine structural and FDG-PET imaging, in order to compare the predictive power of multimodal to unimodal models. Studies utilizing FDG-PET have shown significant predictive ability in the prediction and progression of disease.

## Figures and Tables

**Table 1.** Classification Results from RVM.

Classification Prediction per group								
	AD	BV	CN	nfPPA	PSP	svPPA	N	TPR
AD	143	20	14	6	7	6	196	0.91
BV	6	42	9	2	5	1	65	0.73
CN	28	5	311	20	47	1	412	0.65
nfPPA	8	8	6	15	6	1	44	0.75
PSP	3	4	8	1	42	0	58	0.34
svPPA	2	2	0	0	0	42	46	0.72

AD = Alzheimer’s Dementia; BV = Behavioral Variant of Frontotemporal Dementia; nfPPA = Non-fluent Primary Progressive Aphasia; PSP = Progressive Supranuclear Palsy; svPPA = Semantic variant Primary Progressive Aphasia; TPR = True Positive Rate. Results show the predicted classification for each group with the diagonal indicating a correct classification.

## ABBREVIATIONS

MRI: magnetic resonance imaging
PET: positron emission topography
AD: Alzheimer’s Disease
FTD: frontotemporal dementia
